# Simulating ComBat: how batch correction can lead to the systematic introduction of false positive results in DNA methylation microarray studies

**DOI:** 10.1186/s12859-020-03559-6

**Published:** 2020-06-30

**Authors:** Tristan Zindler, Helge Frieling, Alexandra Neyazi, Stefan Bleich, Eva Friedel

**Affiliations:** 1 Department of Psychiatry, Social Psychiatry and Psychotherapy, Hannover Medical School, Hannover, Germany; 2 Department of Psychiatry and Psychotherapy, Charité Campus Mitte (CCM), Charité-Universitätsmedizin Berlin, Berlin, Germany; 3 Berlin Institute of Health (BIH), 10178 Berlin, Germany

**Keywords:** DNA methylation, Simulation, EPIC array, 450 K array, Illumina, Batch effects, ComBat

## Abstract

**Background:**

Systematic technical effects—also called batch effects—are a considerable challenge when analyzing DNA methylation (DNAm) microarray data, because they can lead to false results when confounded with the variable of interest. Methods to correct these batch effects are error-prone, as previous findings have shown.

**Results:**

Here, we demonstrate how using the R function ComBat to correct simulated Infinium HumanMethylation450 BeadChip (450 K) and Infinium MethylationEPIC BeadChip Kit (EPIC) DNAm data can lead to a large number of false positive results under certain conditions. We further provide a detailed assessment of the consequences for the highly relevant problem of *p*-value inflation with subsequent false positive findings after application of the frequently used ComBat method. Using ComBat to correct for batch effects in randomly generated samples produced alarming numbers of false discovery rate (FDR) and Bonferroni-corrected (BF) false positive results in unbalanced as well as in balanced sample distributions in terms of the relation between the outcome of interest variable and the technical position of the sample during the probe measurement. Both sample size and number of batch factors (e.g. number of *chips*) were systematically simulated to assess the probability of false positive findings. The effect of sample size was simulated using *n* = 48 up to *n* = 768 randomly generated samples. Increasing the number of corrected factors led to an exponential increase in the number of false positive signals. Increasing the number of samples reduced, but did not completely prevent, this effect.

**Conclusions:**

Using the approach described, we demonstrate, that using ComBat for batch correction in DNAm data can lead to false positive results under certain conditions and sample distributions. Our results are thus contrary to previous publications, considering a balanced sample distribution as unproblematic when using ComBat. We do not claim completeness in terms of reporting all technical conditions and possible solutions of the occurring problems as we approach the problem from a clinician’s perspective and not from that of a computer scientist. With our approach of simulating data, we provide readers with a simple method to assess the probability of false positive findings in DNAm microarray data analysis pipelines.

## Background

In the last two decades, the field of epigenetics has opened up new perspectives on complex medical questions [1–3]. DNA methylation (DNAm) is assumed to be modulated both by heritable factors [4] and by environmental conditions [5, 6]. DNAm has received considerable attention in the field of epigenetics research as a source of potential disease-related biomarkers and as one of the missing translational links between nature and nurture [7, 8]. Despite numerous successful findings of disease related biomarkers [2, 8, 9] and the enthusiasm within this novel and promising field of study, it is important to obtain a more nuanced picture of the biological processes related to the biomarkers being assessed, and to develop a more comprehensive and commonly accepted framework for their analysis. At present, researchers in this field have to cope with new technological possibilities and an insufficient understanding of the methylome at the same time [4, 10, 11].

**Fig. 1:**
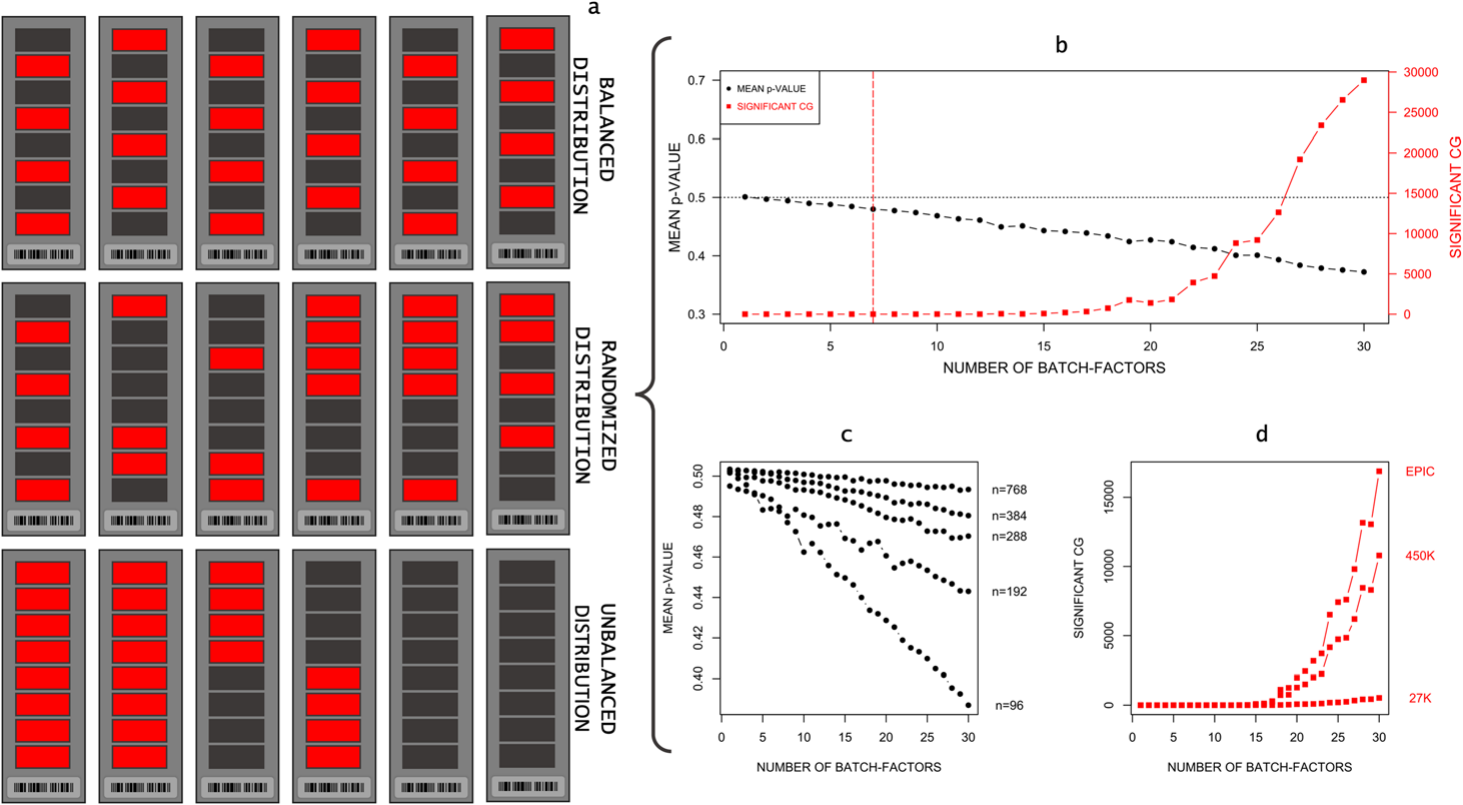
**a** Different case and control sample distributions of *n* = 48 on six chips (red, case; dark grey, control). **b** Systematic increase of the number of factor levels for one corrected factor. The vertical red line shows the factor level with the first false FDR-significant result. **c** Mean p-value decrease for different sample sizes in combination with a systematic increase of the number of factor levels. **d** Number of FDR-significant CpG sites with a systematic increase of factor levels for the different Illumina arrays

Microarray platforms such as the Infinium BeadChips have played a big role in making cost-effective genome-wide measurements of DNAm possible. However, while these platforms can be used to investigate methylation sites across the genome, they assay less than 4% of the CpG methylation sites in the human genome [12] and subsequently confront researchers with numerous study design pitfalls: While the large number of measurement points (up to 850,000 on the Infinium MethylationEPIC BeadChip Kit) results in “big data”, the number of measured samples often remains relatively small. This disproportionate number of samples relative to the number of measurement points represents a massive challenge for subsequent statistical analyses [13]. Another challenge is caused by so-called “batch effects”, which include a series of effects caused by technical variability due to the time, place, and materials used (*batches*). Batch effects are almost impossible to avoid: This is because the individual samples are measured on *chips* - in case of an Infinium MethylationEPIC BeadChip Kit array - with 8 individual samples on each *chip*. These samples are arranged in *rows* at distinct positions (*sample wells*) on the *chips*. The *chips* in turn are mounted on a plate (*samples plate*) that has space for 12 chips (96 samples). The term “batch effect” is referring to the systematic measurement errors between the *sample wells* in *rows*, the *chips* and the *samples plates*. Additional batch effects can be caused for instance by different times of measurement, different sample handling, different sites in multicenter studies and possible undiscovered effects due to the novelty of the technologies [14]. Because they are linked to the materials used to measure the samples, the batch factor number increases linearly with each additional sample (e.g. for each *chip*). Batch effects can dramatically reduce the accuracy of measurements [14] and can produce false positive effects if the sample distribution during the measurements is not uniformly distributed with regards to the *outcome of interest* (*unbalanced sample* – Fig. 1a). This can lead to significant group differences caused by measurement errors being wrongly attributed to the outcome of interest [15, 16].

To make matters even more complicated, there is no validated and commonly accepted framework for the analysis of genome-wide epigenetic data. Based on this lack of consistency in data analyses, there is little empirical knowledge about what outcomes to expect, which makes it extremely difficult for researchers to assess and replicate new findings [11].

With the R package *ChAMP* [17], an important attempt has been made to integrate the different steps necessary for the analyses of Infinium HumanMethylation450 BeadChip (450 K) and Infinium MethylationEPIC BeadChip Kit (EPIC) data into a comprehensive analysis pipeline. *ChAMP* addresses the problem of batch effect correction with the ComBat method [18], which uses an empirical Bayesian approach to avoid over-correction—a critical feature to use with small sample sizes. ComBat has been heavily praised as being the most effective method for counteracting batch effects [19] when they are known [20]. It is implemented in the *sva* package [21], which itself is integrated into the *ChAMP* pipeline.

While ChAMP offers only a few settings for ComBat, the direct sva function call offers more options for using ComBat. Among other options there is the possibility to specify a model matrix being used *(“mod”)* with covariates or a *outcome of interest* variable besides the batch factors. Champ automatically passes the *outcome of interest variable* to ComBat.

In an earlier study with 69 subjects, we found a high number of CpG-sites with significantly differing methylation levels between two groups using the *ChAMP* analysis pipeline. When we used the ComBat method for batch correction provided in *ChAMP* to correct for *row* and *chip batches*, we became aware of previously reported problems with this approach: Two cautionary case reports were published in 2018 [22] and in 2014 [23] reporting problems experienced when ComBat was used to correct for batch effects in *450 K* data. After applying ComBat to adjust for a non-biological signal, Prince and Robinson reported that roughly 10 k to 20 k significant CpG sites (false discovery rate (FDR) < 0.05) emerge. These sites had not been present before ComBat correction and were not replicable under a revised analysis design and use of ComBat. In an analogous way in an earlier report, Buhule found 25 k differentially methylated CpG-sites (FDR < 0.05) before batch correction, but around 100 k significant CpG-sites after correction. Both studies analyzed data from pilot studies with limited sample sizes (*n* = 30 in [22]; and *n* = 92 in [23]) and used the Illumina *450 K* array. While these case reports should warn researchers from blindly applying ComBat or similar methods to remove batch effects from an unbalanced sample, it remains unclear to what extent this effect occurs and how this effect relates to varying sample sizes and to the different Illumina arrays. Furthermore, as Price and Robinson aptly stated: “[ …] it is alarming that thousands of false discoveries might have been claimed if the analysis had been limited to standard processing pipelines”.

In this report, we aim to provide a detailed assessment of consequences of applying the frequently used ComBat method for the highly relevant problem of *p*-value inflation, which results in subsequent false positive findings [18]. We use this approach as a hands-on example of how to systematically investigate the methods used in multi-step microarray platform analysis pipelines.

We further provide researchers with a simulation-based quantification of ComBat-introduced false signal induction under various configurations, and a simple tool to assess the probability of false positive findings in DNAm microarray data analysis pipelines.

## Methods

As clinicians, we have become increasingly aware of the problems associated with using multi-step analysis pipelines without being able to realistically verify the source code of all the methods used. This has led us to the conclusion that a simulation should be performed to investigate these problems, without the interference of possible real biological signals. Our simulation was created using the packages discussed, namely *sva* [14] and *ChAMP* [17], which are implemented in the R (3.6.1) and Bioconductor (3.9) environments on Windows 10.0.18362.

The basis for our simulation were 758,289 mean and standard deviation values from probes on an *EPIC* array, based on data from 69 patients collected in our laboratory.^1^

Based on this data, random numbers were generated from a normal distribution using the “rnorm” function with the “Mersenne-Twister” algorithm [24] to generate simulated methylation beta values for every CpG site with a mean and standard deviation corresponding to the natural CpG sites.^2^ Data from our laboratory were not used in any further analyses.

The data generated in this way cannot contain any signal, and therefore cannot contain any batch effect either. In our opinion, this is the best way to investigate the effect of ComBat on the data. The alternative approach of investigating the effects of ComBat on real data would always carry the risk of producing true positive results, which would therefore make an accurate analysis of false positive findings much more difficult.

The resulting data from our simulation share many properties with their biological CpG counterparts, such as the distribution of differences in type-I and type-II CpG-sites which occur in Illumina arrays for technical reasons [25].^3^ This enabled us to follow the *ChAMP* pipeline as planned and in the next step to normalize the data for type-I and type-II differences, as required by the manual for the *sva* package [21] using the beta-mixture quantile normalization (BMIQ) method [26]. BMIQ serves as an intra-sample normalization procedure, correcting the deviation of type-II probe values, and is implemented within the “champ.norm” function.

Because it can be argued that a test without batch effects is not very naturalistic and offers the risk of being a special case, we added in a second step artificial batch effects to our data. For this purpose, we based the batch effect simulation on the preliminary work of Wen Bin Goh and Wong [15] by adding randomly generated effects between 1% and − 1% to the previously generated data. This intended to simulate a simple technical brightness offset during measurement. Each sample was added with the respective systematic errors for *row* and each *chip*.

Following this, the basic effects of *balanced*, *unbalanced*, and *random sample distributions* were tested on 48 randomly generated samples. Testing was applied with and without batch effects and with 100 simulation repetitions each. This part of our analysis set the starting point for our simulations. It was performed using the “champ.runCombat” function, as well as with a direct call to the function provided within in the *sva* package, while the subsequent analyses used the “champ.runCombat” function. The function provided by the *sva* package was additionally executed with and without the use of a “model matrix for *outcome of interest* and other covariates besides batch” (*mod*) option [14].

After this basic analysis, effects of ComBat were systematically tested for *randomly* distributed samples under varying conditions. We consider the *balanced* and *unbalanced sample distributions* to be the exceptions, with the random sample distribution the normal case for most studies. Accordingly, the functions provided by the *ChAMP* pipeline were encapsulated in simulation loops.

At first, *n* = 96 simulated samples—corresponding to size of one *samples plate*—were used to gradually increase the number of ComBat-processed factor levels. In a second simulation, the number of batch factors was increased with two equal-sized randomly distributed factor levels each. In a second simulation, we investigated the effect of increasing the sample size in five steps from *n* = 96 (one *samples plate* / 8 *chips*) up to *n* = 768 samples. After these simulations with respect to the Illumina *EPIC* array containing 850,000 CpG-sites, the effects of ComBat on older and smaller arrays (Illumina Infinium HumanMethylation27 BeadChip (*27 K)* and *450 K*) with *n* = 96 samples were examined. *27 K* and *450 K* data were created by sampling from the *EPIC* data accordingly.

As a final analysis a simulated sample of *n* = 48 normal distributed probes were enriched with 2000 CpG sites with significant/trend wise (uncorrected) group differences with respective *p*-values from 10^− 1^ to 10^− 20^. Following this step, the Dataset again was added with systematic batch effects for *row* and *chip.*

In all simulations, the average *p*-value and the number of significant CpG-sites after FDR correction were analyzed with the “champ. DMP” function. This function uses the *limma* package to calculate the *p*-value for differential methylation by applying a linear model. If ComBat batch correction works as expected, neither an increase of significant CpG-sites nor a deviation from a mean *p*-value of 0.5 would be expected for the normally distributed data without simulated batch effects. In the first simulation with and without simulated batch effects, the *p*-value distribution was additionally analyzed using Q-Q plots and using the genomic inflation factor λ for all corresponding sample distributions and ComBat variants. The genomic inflation factor λ is defined as the ratio between the medians of the ComBat-corrected distribution of the test statistic and the expected statistic without ComBat correction. This therefore quantifies the magnitude of the bulk inflation and of the excess false positive rate [27].

In order to validate the results of our systematic simulations relative to real research results, we simulated the preliminary results of two studies [22, 23] according to their factor structure using our *ChAMP*-based simulation.

## Results

While researchers have previously suspected that unwanted effects would only occur in unbalanced study designs [22, 23, 28], our simulation of *n* = 48 samples over 100 simulation runs showed^4^ a considerable undesired effect of ComBat on all sample distributions (Fig. 1a). The test statistics were examined using the mean *p*-value and λ, which both showed considerable deviations from their expected values (expected: mean *p* = 0.5; *λ* = 1) for all variants and distributions.^5^ The smallest deviation from the expected values was obtained by simulating the random sample distribution in conjunction with ComBat, which was implemented by the *sva* package without the *“mod”* option enabled (mean *p* = 0.49; *λ* = 1.11). In accordance with the previously published reports, the *unbalanced* sample distribution (Fig. 1a) showed the highest simulated deviation using the ComBat implementation of *ChAMP* (mean *p* = 0.31; *λ* = 3.26), resulting in *M*_FDR_ = 109,097 FDR–significant and *M*_BF_ = 1293.38 Bonferroni-corrected (BF) CpG sites. In comparison to these results, the sampled random distribution (Fig. 1a) showed much smaller distortion of the test statistics (mean *p* = 0.41; *λ* = 1.81), resulting in *M*_FDR_ = 5597.48 FDR-significant and *M*_BF_ = 23.81 BF false significant sites. As expected, the balanced sample design showed the smallest *p*-value reduction (mean *p* = 0.41; *λ* = 1.72). However, contrary to previous reports, the observed impact of ComBat on the sample was enough to yield *M*_FDR_ = 3159.64 FDR (*M*_BF_ = 16.06) false significant CpG sites. These effects could be replicated in comparable sizes for the *sva* package implementation of the ComBat function with the *“mod”* option enabled^4^. While all the simulated distortions observed were in the direction of an enhanced significance, the opposite effect was observed in the *sva* package implementation using the unbalanced sample distribution without the target variable option (mean *p* = 0.58; *λ* = 0.41). While this substantial deviation did not generate any false significant CpG sites, it could in theory result in a substantially elevated number of false negative sites. While all the simulated test statistics showed undesirable deviations, the resulting number of false significant sites was negligible for the random and unbalanced sample distributions when the *sva* package implementation without the *“mod”* option was used. Any other combination resulted in a substantially elevated number of false significant CpG sites. It is important to note that for this variant of ComBat, the balanced sample distribution generated a considerable number of false significant sites, with up to 55 FDR-significant sites.

**Fig. 2:**
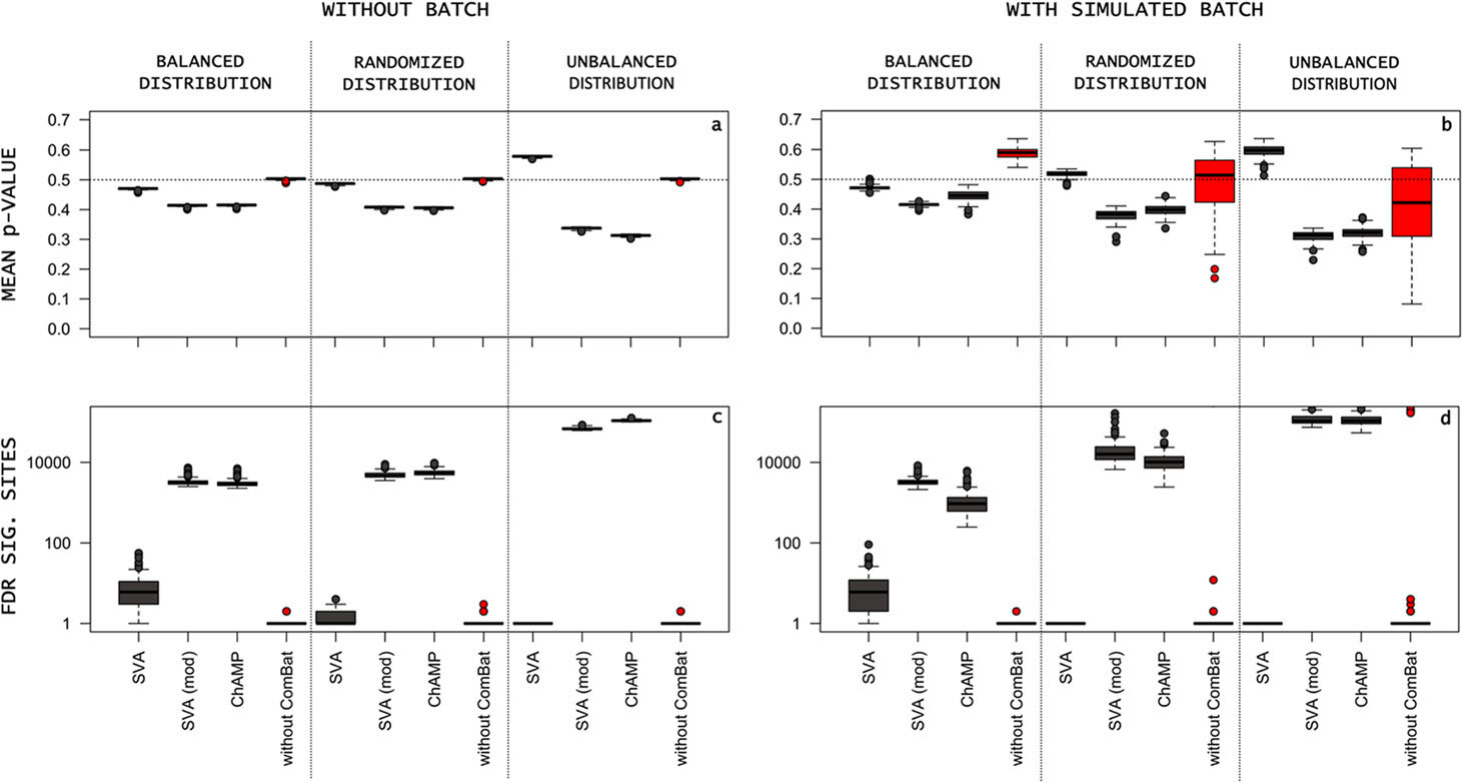
“mod” refers to a model matrix for the outcome of interest. **a** Boxplots of *p*-value distributions without added batch effects and under various conditions. Dotted line indicates expected mean *p*-value of 0.5. **b** Boxplots of *p*-value distributions with simulated batch effects and under various conditions. **c** Boxplots of FDR false significant sites without batch. **d** Boxplots of FDR false significant sites without batch. Boxplots for “*without ComBat*” are indicating the false positive sites due to uncorrected batch effects for the respective sample distributions

The corresponding simulation with added simulated batch effects^6^ showed the same trends concerning the relative distortion of the mean *p*-values (Fig. 2c). In addition to investigating the effect of ComBat, Fig. 2d (red) shows the potential for false positive results due to uncorrected batch effects: The uncorrected, unbalanced simulation with batch effects showed a very high potential of false FDR significant CpG (*M* = 41,912.5, *SD* = 111,629.7) due to batch effects.

A subsequent systematic investigation of ComBat-introduced effects (based on the ComBat implementation in *ChAMP*) showed a considerable effect of ComBat on the results—the mean *p*-value decreased as the number of batch factor levels increased (Fig. 1b) and as the number of batch factors increased. The first FDR- and BF-significant CpG sites appear at a batch factor number of 7 and these increase exponentially in number with increasing factor levels from this point on. Using this approach, we are able to demonstrate that even a small number of factor levels or factors can lead to a considerable number of false positive FDR- or even BF-corrected results. The number of such FDR-corrected significant sites increases exponentially with the number of factor levels.

A systematic simulation of the influence of sample size (Table 1) on the reported effects showed that an increase in sample size reduced the decrease in mean *p*-value, but that this effect was nevertheless detectable in every sample size (Fig. 1c). Using correction for the row and chip with a random sample distribution resulted in 118 FDR-significant sites at a sample size of *n* = 768. For the balanced sample, this resulted in a consistent reduction in the number of false positive sites with increasing sample size, whereby false positives were not observed until the sample size reached *n* = 786. For the unbalanced sample distribution, however, the exact opposite process was observed, with a steady worsening of the deviation of the test statistic, resulting in an extreme inflation of the number of significant sites, with up to 500 K FDR-significant sites.

**Table 1:** ComBat (ChAMP) applied to increasing sample sizes

sample size	batch factors	balanced				random				unbalanced			
		mean-*p*	λ^+^	FDR*	BF**	mean-*p*	λ^+^	FDR*	BF**	mean-*p*	λ^+^	FDR*	BF**
*n* = 48	8 rows + 6 chips	0.4131827	1.720126	3540	15	0.3735538	1.990719	27,057	132	0.2872	3.880235	156,084	2803
*n* = 96	8 rows + 12 chips	0.4395739	1.465350	265	4	0.4254060	1.538365	1412	19	0.2609	4.999723	214,009	11,048
*n* = 144	8 rows + 18 chips	0.4487983	1.391675	84	2	0.4308005	1.453891	1218	8	0.2335	6.362690	273,773	25,522
*n* = 192	8 rows + 24 chips	0.4533012	1.355385	47	6	0.4438228	1.428494	218	2	0.2133315	7.746447	318,618	42,427
*n* = 240	8 rows + 30 chips	0.4565824	1.334509	27	1	0.4424769	1.419485	226	4	0.1978271	9.738140	352,805	59,949
*n* = 288	8 rows + 36 chips	0.4588096	1.320805	9	2	0.4443718	1.406706	181	4	0.1854536	11.319961	379,740	77,903
*n* = 336	8 rows + 42 chips	0.4580821	1.310937	11	2	0.4453380	1.388399	144	4	0.1733198	13.128076	406,856	97,847
*n* = 384	8 rows + 48 chips	0.4589804	1.303460	28	1	0.4512571	1.370874	41	3	0.1574975	15.871418	440,828	127,703
*n* = 432	8 rows + 54 chips	0.4609161	1.296584	2	0	0.4532645	1.360342	84	4	0.1565864	16.385741	442,872	130,896
*n* = 480	8 rows + 60 chips	0.4629813	1.290615	0	0	0.4545829	1.352150	77	3	0.1495949	18.068155	457,389	146,684
*n* = 528	8 rows + 66 chips	0.4640470	1.286172	4	1	0.4541513	1.347141	89	4	0.1444482	19.435062	469,246	159,929
*n* = 576	8 rows + 72 chips	0.4643993	1.282560	5	0	0.4562415	1.346690	67	3	0.1392911	21.116253	480,228	174,133
*n* = 624	8 rows + 78 chips	0.4647209	1.279449	1	1	0.4546367	1.351416	97	3	0.1319790	22.583813	495,269	193,145
*n* = 672	8 rows + 84 chips	0.4646304	1.277031	10	0	0.4550306	1.344902	99	1	0.1285275	24.769395	501,777	201,233
*n* = 720	8 rows + 90 chips	0.4629008	1.275070	5	1	0.4552286	1.342806	94	3	0.1250251	26.836670	509,759	213,140
*n* = 768	8 rows + 96 chips	0.4631314	1.273107	0	0	0.4545927	1.341437	118	3	0.1209156	28.445529	517,941	224,289

^+^ Genomic Inflation Factor λ

^*^ Significant CpG sites with False Discovery Rate 5%

^**^ Significant CpG sites with Bonferroni correction 5%

Comparison between *27 K*, *450 K*, and *EPIC* results showed a similar picture for the *EPIC* and *450 K* array sizes, but a considerably smaller effect on *27 K* array size (Fig. 1d).

A close examination of the false significant CpG sites introduced showed that neither extreme values (close to 0 or 1) nor outliers played a role in the creation of false significant CpG sites. It was primarily the CpG sites showing randomly assigned high group differences before ComBat correction that were affected. A closer look at the individual values showed that all values were slightly changed and that this change was not evenly distributed across both groups.^7^

**Fig. 3:**
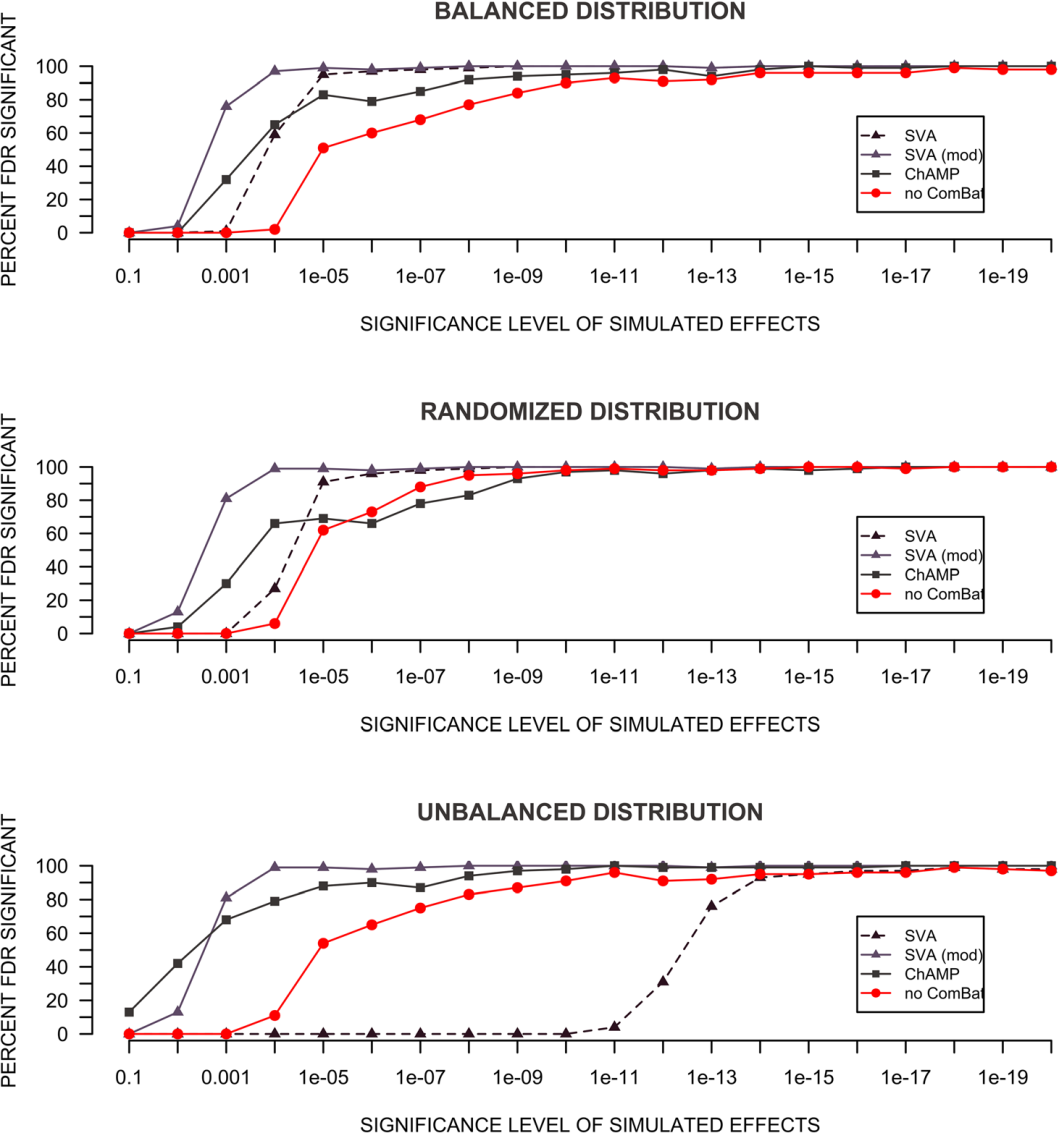
Detection auf added significant effects for different use cases of ComBat. The X axes show the uncorrected p-value of the simulated effects before adding the batch effects. The Y axes show the percentage of CpG sites detected as FDR *p* < 0.05 after adding the batch effects for the different forms of batch effect correction

The analysis of simulated significant/trend wise (uncorrected) group differences showed that ComBat in all his variants has a positive effect on the detection of significant differences (Fig. 3). An exception is the application of Combat (SVA without *mod*) to an *unbalanced* sample. Here the detection of significant sites is strongly deteriorated, which is in strong correspondence with the results of the previous simulations (Fig. 2a), where an increased mean *p*-value has been shown for this use case.

Next, we validated our findings with a simulation based on data from real studies: Price and Robinson [22] found 9612 differentially methylated CpG sites in their first comparison, and 19,214 sites in their second comparison. A simulated result with 50 runs yielded *M* = 11,270.74 (*SD* = 221.70) differentially methylated CpG sites for the first comparison, and *M* = 23,477.06 (*SD* = 382.67) for the second comparison.

For the factor structure of Buhule et al. [23], our simulation with 50 runs predicted *M* = 103,188.70 (*SD* = 481.84) differentially methylated CpG sites, while 94,191 false significant CpG sites were reported in the study.

## Assessment of ComBat in previously published empirical studies

Because of the potential implications of our results, we conducted a systematic search for previously published empirical studies. We used the full text search provided in Google Scholar to successfully identify studies which employed ComBat in their statistical analysis. Using this approach, we were able to access 54 papers published since 2018 that used *Illumina 450 K* and *EPIC* arrays in combination with ComBat for their analyses. Unfortunately, none of the identified authors provided sufficient detail regarding their batch factor structure, sample distribution, and the subsequent application of ComBat to enable us to recreate their complete analyses sufficiently. 72.73% of authors merely indicated that ComBat was used, but not to what extent, while the remaining studies specified the corrected factors, but were missing the exact number of factors or did not specify the sample distribution. Some authors tried to mitigate the inflation in the occurrence of significant sites by reporting only those CpG sites which had a minimum methylation rate difference between groups.

## Discussion

When technical factors related to DNAm analyses, such as the *chips*, the position of the samples on the *chips*, or even the date of processing, are confounded with the dependent variable of interest, this can very easily lead to disastrously false results (Fig. 2d) [29, 30]. However, as indicated above, the use of batch effect reduction methods can potentially lead to equally large problems. The results of our simulation study reveal a fundamental problem: in addition to the impossibility of identifying significant effects after correction for multiple testing with small sample sizes, it is just as impossible with increasing sample sizes to avoid severe batch effects due to technical (e.g. *samples pl*ate and *chip* size) or practical (multicentric studies) reasons.

Moreover, our results show that the use of ComBat for batch effect mitigation can lead to any number and magnitude of false significant results, which occur in a variety of different use cases. We were able to successfully replicate the results of the earlier studies by Buhule et al. and by Price and Robinson [22]. The results reported here strongly support the important warnings made by other authors to double-check every step of a DNAm analysis and to be skeptical about the results obtained using ComBat for batch effect mitigation on unbalanced samples [22, 23, 28, 31]. Furthermore, our results imply that these warnings should be extended to balanced samples too. At this point it is important to note that in our results the combination of a *randomized* sample distribution and ComBat usage without the use of covariates or a *outcome of interest* variable was least prone to error. This is also the usage variant shown in the official tutorial of sva, even if the manual of sva suggests other possible use cases. However, in reality the use of ComBat often seems to be a different one due to seemingly better results as the integration in ChAMP suggests.

Furthermore, the positive effect on the actual removal of batch effects with ComBat and the subsequent better detection of actual real effects can be replicated (Fig. 3) in accordance with previous studies [22, 23].

**Fig. 4:**
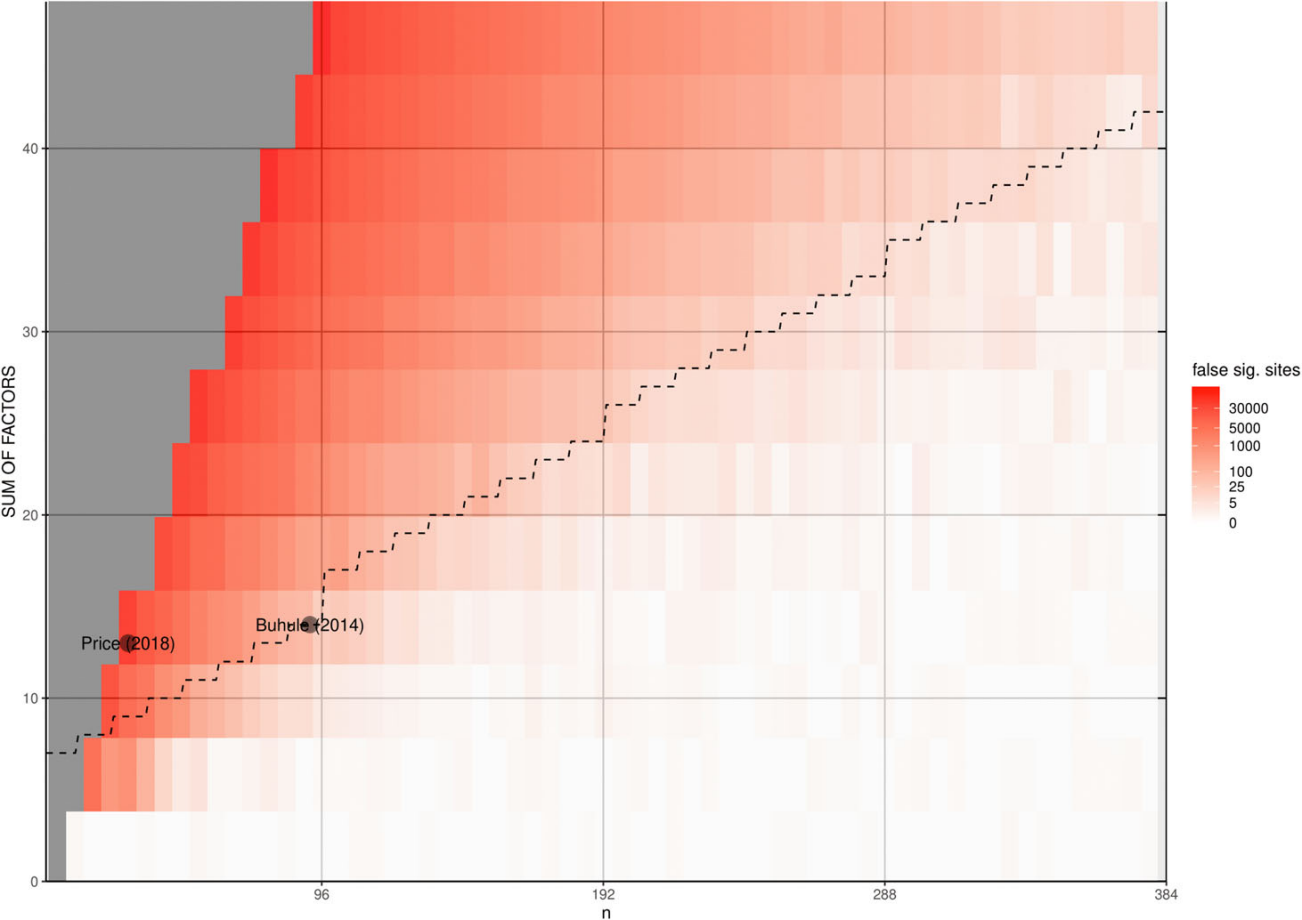
Heatmap of FDR-significant CpG sites after ComBat application. The dotted line indicates the number of corrected factors when “correcting” for a technical batch. Grey tiles were not assessed due to the sample–factor ratio. The redder the cells are, the more false significant CpG sites were found after applying ComBat

Unfortunately, the precise extent of the consequences of our simulation results remains unclear, since most authors do not report their correction methods in detail. Because of this, we were unable to confirm the results of other previously published studies which used ComBat. Therefore, we created Fig. 4, which is intended to provide a rough guideline for evaluating studies. It is provided without any guarantee of correctness, because a precise evaluation would depend on the exact factor structure and ComBat configuration used by the study. We therefore appeal to authors to describe their use of analysis pipelines in greater detail and to provide all of the information necessary to replicate a finding. In this case, the R and Jupyter Notebooks might be a good starting point, because they are capable of combining theoretical considerations with the practical implementation.

The limitations of this study were that the effects presented were restricted to the use of ComBat, which is only one (however frequently used) possible method out of many for correcting for batch effects in whole methylome data. The simulation of effects applied here needs to be further validated using real data and can only be interpreted as an approximation of the real effects. Additionally, the results presented here are limited to probe-wise (DMP) analyses, because the naturally occurring intercorrelation of CpG sites is not accounted for in our simulation approach.

Furthermore, it is important to note that our batch effect simulation was very basic. It is plausible that many different and more complex batch effects exist, which are not sufficiently covered by this study and could potentially produce different results. Therefore, it is important that the structure of batch effects itself is analyzed as accurately as possible in further research.

## Conclusions

Even if our work suggests otherwise at first glance, ComBat can be a very good method for batch effect removal. But it is crucial not to use this method blindly and to be very skeptical of positive findings that cannot be found without correction. In this context we want to emphasize the importance of the sample distribution. The distribution of the samples during the measurement is fundamental for the creation of batch effects and, as we have shown, their removal. This is why the distribution of samples should be presented in full detail in future studies. We want to recommend visual illustrations like Fig. 1a following the example of Buhule et.al [23]. for this purpose. Furthermore, contrary to previous research results, we can only advise against using any kind of non-random sample distribution. Our simulation suggests that balanced samples with respect to the *outcome of interest* pose new problems that have been underestimated in the literature so far.

While in our view, efforts to integrate and standardize DNAm analysis in packages such as *ChAMP* are an essential step toward extending the replicability and comparability of results, it must be noted that this integration means that *ChAMP* alone depends on 233 separate packages. Therefore, it is impossible in practice for researchers to check all of these packages with their respective source codes for correctness and correct usage. This is particularly problematic because statistical hypothesis testing (“*p*-value statistics”) only works correctly if all requirements for these methods are met in the pre-processing and the structure of the data. While the results of our simulations do not allow conclusions to be made about other correction methods, similar problems are possible with other methods. This means that a simple method of verifying results is required. Therefore, we would encourage researchers to apply our deliberately simple yet effective method for checking for false positive results to their own analysis pipelines using the R code provided in the Supplementary Material.

Summarizing the lessons learned from our results, we strongly recommend not blindly trusting the analysis pipelines discussed above. Testing them with random data without the possibility of real significant results is a simple way to test the pipelines and sample distributions in advance. In addition, the scientific community urgently needs to develop a standardized way to adequately present complex statistical analysis methods.

## Supplementary information

**Additional file 1.** Supplementary Document with two sections: A. DNA methylation quantification and quality control. B. Assessment of the simulated data. Methylation Simulation Script. Contains all descriptive data necessary for the simulation as well as simple R code to create any number of simulated *EPIC* methylomes.

**Additional file 2: Supplementary Figure S1.** Q-Q plots of simulation run 1 with 48 samples.

**Additional file 3: Supplementary Figure S2.** Scatterplot of one false significant CpG site from simulation run 1 with and without application of ComBat.

**Additional file 4: Supplementary Table S1.** Full report of the simulation results with *n* = 48 without simulated batch effects

**Additional file 5: Supplementary Table 2.** Full report of the simulation results with *n* = 48 with simulated batch effects.

## Data Availability

All datasets for this study are included in the manuscript and in the supplementary files.

## Abbreviations

DNAm: DNA methylation
450 K: Infinium HumanMethylation450 BeadChip
27 K: Illumina Infinium HumanMethylation27 BeadChip
BF: Bonferroni-corrected
BMIQ: Beta-mixture quantile normalization
EPIC: Infinium MethylationEPIC BeadChip Kit
FDR: False discovery rate
mod: Possibility to specify a model matrix being used with covariates or a outcome of interest variable besides the batch factors in the ComBat implementation of the SVA package

## Footnotes

1 More information about laboratory data DNA methylation quantification and quality control is available in **Supplementary Document - Section A**

2 Data and the R code for the simulation are available as a Supplementary Material (MethylationSimulationScript.ZIP)

3 A more thorough description of the simulation and the resulting simulated value distributions can be found in **Supplementary Document – Section B**

4 **Supplementary Table 1** provides a full report of the simulation results

5 **Supplementary Figure 1** provides Q-Q plots for test statistics

6 **Supplementary Table 2** provides a full report of results from the simulation with added batch effects

7 **Supplementary Figure 2** provides a scatterplot of a false significant CpG site before and after ComBat correction.
