# Supplementary figures and images for: Simulating ComBat: how batch correction can lead to the systematic introduction of false positive results in DNA methylation microarray studies
Source: BMC Bioinformatics. 2020 Jun 30;21:271. doi: 10.1186/s12859-020-03559-6 (PMC7328269; doi:10.1186/s12859-020-03559-6)

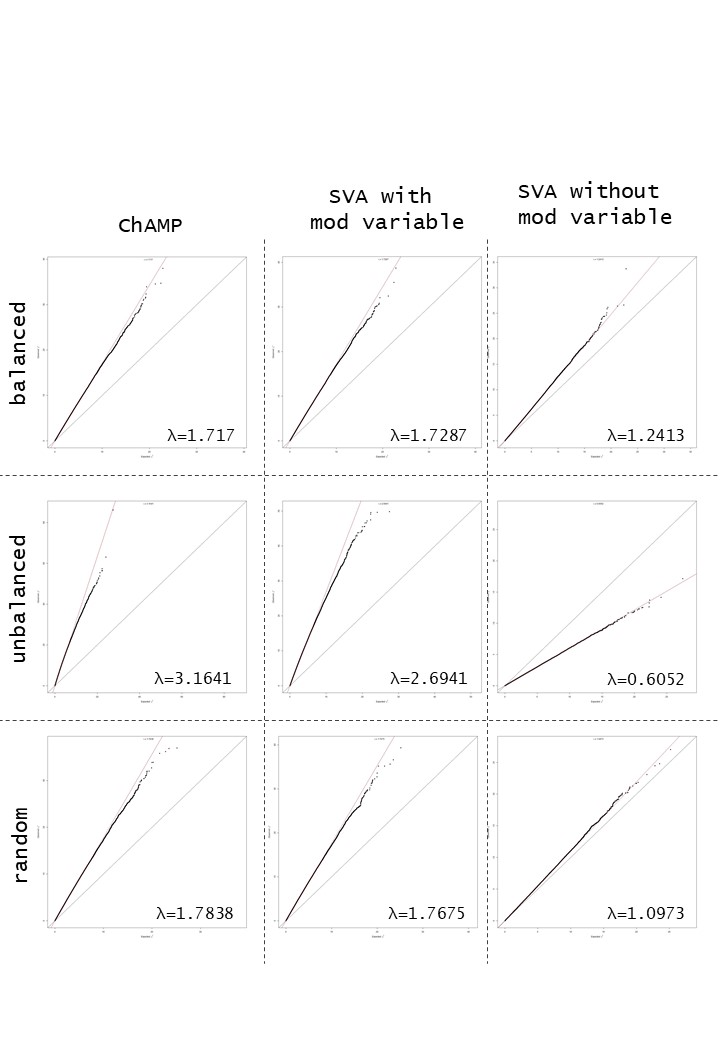

Supplement: Supplementary file 2 — Additional file 2: Supplementary Figure S1. Q-Q plots of simulation run 1 with 48 samples. [file 12859_2020_3559_MOESM2_ESM.jpg]

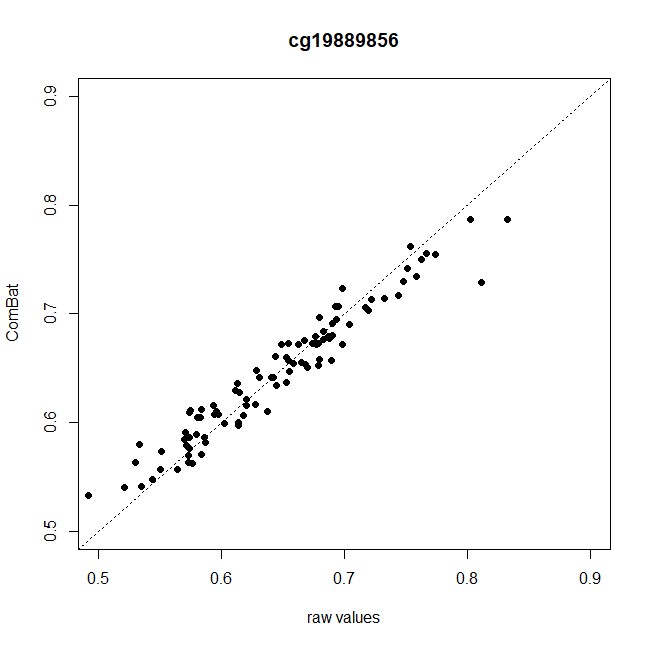

Supplement: Supplementary file 3 — Additional file 3: Supplementary Figure S2. Scatterplot of one false significant CpG site from simulation run 1 with and without application of ComBat. [file 12859_2020_3559_MOESM3_ESM.jpg]
