# Supplementary material for: Simulating ComBat: how batch correction can lead to the systematic introduction of false positive results in DNA methylation microarray studies
Source: BMC Bioinformatics. 2020 Jun 30;21:271. doi: 10.1186/s12859-020-03559-6 (PMC7328269; doi:10.1186/s12859-020-03559-6)
Supplement: Supplementary file 4 — Additional file 4: Supplementary Table S1. Full report of the simulation results with n = 48 without simulated batch effects [file 12859_2020_3559_MOESM4_ESM.pdf]

Supplementary Table 1 – Full report of the simulation results without simulated batch effects

100 simulation runs with n=48 samples

	balanced				random				unbalanced			
	mean-*p*	λ^+	FDR*	BF**	mean-*p*	λ^+	FDR*	BF**	mean-*p*	λ^+	FDR*	BF**
ComBat (SVA) without *mod*++	*M*=0.4694<br>*SD*=0.0035	*M*=1.2424<br>*SD*=0.0027	*M*=7.71<br>*SD*=9.87<br>min=0<br>max=55	*M*=1.30<br>*SD*=1.06<br>min=0<br>max=4	*M*=0.4869<br>*SD*=0.0029	*M*=1.1113<br>*SD*=0.0065	*M*=0.38<br>*SD*=0.69<br>min=0<br>max=3	*M*=0.30<br>*SD*=0.52<br>min=0<br>max=2	*M*=0.5781<br>*SD*=0.0027	*M*=0.6133<br>*SD*=0.0065	*M*=0<br>*SD*=0<br>min=0<br>max=0	*M*=0<br>*SD*=0<br>min=0<br>max=0
ComBat (SVA) with *mod*++	*M*=0.4131<br>*SD*=0.0034	*M*=1.7282<br>*SD*=0.0036	*M*=3397.72<br>*SD*=916.07<br>min=2509<br>max=7375	*M*=16.91<br>*SD*=4.66<br>min=7<br>max=31	*M*=0.4075<br>*SD*=0.0029	*M*=1.7879<br>*SD*=0.0112	*M*=4939.89<br>*SD*=988.39<br>min=3550<br>max=9009	*M*=21.62<br>*SD*=5.98<br>min=8<br>max=41	*M*=0.3370<br>*SD*=0.0034	*M*=2.7773<br>*SD*=0.0552	*M*=68913.91<br>*SD*=4982.63<br>min=61438<br>max=85181	*M*=503.57<br>*SD*=69.22<br>min=407<br>max=754
ComBat (ChAMP)	*M*=0.4143<br>*SD*=0.0034	*M*=1.7165<br>*SD*=0.0036	*M*=3159.64<br>*SD*=886.81<br>min=2271<br>max=7033	*M*=16.06<br>*SD*=4.55<br>min=7<br>max=28	*M*=0.4053<br>*SD*=0.0029	*M*=1.8119<br>*SD*=0.0131	*M*=5597.48<br>*SD*=1056.49<br>min=3939<br>max=9687	*M*=23.81<br>*SD*=6.23<br>min=8<br>max=47	*M*=0.3126<br>*SD*=0.0683	*M*=3.2653<br>*SD*=0.0684	*M*=109097.83<br>*SD*=5594.35<br>min=100391<br>max=126782	*M*=1293.38<br>*SD*=140.28<br>min=1078<br>max=1851
Without ComBat	*M*=0.5023<br>*SD*=0.0035		*M*=0.04<br>*SD*=0.19<br>min=0<br>max=1	*M*=0.04<br>*SD*=0.19<br>min=0<br>max=1	*M*=0.5026<br>*SD*=0.0027		*M*=0.11<br>*SD*=0.34<br>min=0<br>max=2	*M*=0.10<br>*SD*=0.30<br>min=0<br>max=1	*M*=0.5026<br>*SD*=0.0028		*M*=0.38<br>*SD*=0.69<br>min=0<br>max=1	*M*=0.3<br>*SD*=0.52<br>min=0<br>max=1

++ Model matrix for outcome of interest

+ Genomic Inflation Factor λ

* Significant CpG sites with False Discovery Rate 5%

** Significant CpG sites with Bonferroni correction 5%
