# Supplementary material for: Simulating ComBat: how batch correction can lead to the systematic introduction of false positive results in DNA methylation microarray studies
Source: BMC Bioinformatics. 2020 Jun 30;21:271. doi: 10.1186/s12859-020-03559-6 (PMC7328269; doi:10.1186/s12859-020-03559-6)
Supplement: Supplementary file 5 — Additional file 5: Supplementary Table 2. Full report of the simulation results with n = 48 with simulated batch effects. [file 12859_2020_3559_MOESM5_ESM.pdf]

Supplementary Table 2 – Full report of the simulation results with simulated batch effects

100 simulation runs with n=48 samples

	balanced				random				unbalanced			
	mean-*p*	λ⁺	FDR*	BF**	mean-*p*	λ⁺	FDR*	BF**	mean-*p*	λ⁺	FDR*	BF**
ComBat (SVA) without *mod*⁺⁺	*M*=0.4718<br>*SD*=0.0066	*M*=1.6830<br>*SD*=0.1044	*M*=8.9<br>*SD*=12.37<br>min=0<br>max=90	*M*=1.2100<br>*SD*=0.1044<br>min=0<br>max=6	*M*=0.5182<br>*SD*=0.0010	*M*=0.9638<br>*SD*=0.2505	*M*=0<br>*SD*=0<br>min=0<br>max=0	*M*=0<br>*SD*=0<br>min=0<br>max=0	*M*=0.5932<br>*SD*=0.0234	*M*=0.4462<br>*SD*=0.1916	*M*=0<br>*SD*=0<br>min=0<br>max=0	*M*=0<br>*SD*=0<br>min=0<br>max=0
ComBat (SVA) with *mod*⁺⁺	*M*=0.4156<br>*SD*=0.0058	*M*=2.3471<br>*SD*=0.1385	*M*=3486.40<br>*SD*=1115.10<br>min=211.30<br>max=8339	*M*=18.11<br>*SD*=5.33<br>min=7<br>max=34	*M*=0.3775<br>*SD*=0.0281	*M*=2.2440<br>*SD*=0.2505	*M*=23004.12<br>*SD*=22965.91<br>min=6661<br>max=164515	*M*=91.75<br>*SD*=158.56<br>min=25<br>max=1185	*M*=0.3084<br>*SD*=0.0183	*M*=2.744<br>*SD*=1.1052	*M*=116301,55<br>*SD*=33699,57652<br>min=73945<br>max=281912	*M*=1555.86<br>*SD*=1555.22<br>min=615<br>max=14930
ComBat (ChAMP)	*M*=0.4440<br>*SD*=0.019	*M*=2.0752<br>*SD*=0.1373	*M*=1204.11<br>*SD*=990.59<br>min=246<br>max=6156	*M*=10.03<br>*SD*=4.17<br>min=3<br>max=26	*M*=0.3966<br>*SD*=0.0178	*M*=0.3966<br>*SD*=0.0178	*M*=11542.92<br>*SD*=7007.60<br>min=2434<br>max=52349	*M*=51.2<br>*SD*=24.54<br>min=18<br>max=185	*M*=0.3199<br>*SD*=0.0219	*M*=2.8676<br>*SD*=1.3157	*M*=113820.47<br>*SD*=33810.66<br>min=53877<br>max=220904	*M*=2155.95<br>*SD*=1740.92<br>min=526<br>max=9531
Without ComBat	*M*=0.5879<br>*SD*=0.0187		*M*=0.01<br>*SD*=0.1<br>min=0<br>max=1	*M*=0.01<br>*SD*=0.1<br>min=0<br>max=1	*M*=0.4840<br>*SD*=0.1011		*M*=2949.66<br>*SD*=29494.88<br>min=0<br>max=294949	*M*=0.10<br>*SD*=0.36<br>min=2<br>max=2	*M*=0.4057<br>*SD*=0.1341		*M*=41912.5<br>*SD*=111629.70<br>min=0<br>max=547084	*M*=6.26<br>*SD*=47.29<br>min=0<br>max=470

⁺⁺ Model matrix for outcome of interest

⁺ Genomic Inflation Factor λ

* Significant CpG sites with False Discovery Rate 5%

** Significant CpG sites with Bonferroni correction 5%
